# Source Apportionment of Particle Bound Polycyclic Aromatic Hydrocarbons at an Industrial Location in Agra, India

**DOI:** 10.1100/2012/781291

**Published:** 2012-04-19

**Authors:** Anita Lakhani

**Affiliations:** Department of Chemistry, Dayalbagh Educational Institute, Agra 282005, India

## Abstract

16 US EPA priority polycyclic aromatic hydrocarbons (PAHs) were quantified in total suspended ambient particulate matter (TSPM) collected from an industrial site in Agra (India) using gas chromatography. The major industrial activities in Agra are foundries that previously used coal and coke as fuel in cupola furnaces. These foundries have now switched over to natural gas. In addition, use of compressed natural gas has also been promoted and encouraged in automobiles. This study attempts to apportion sources of PAH in the ambient air and the results reflect the advantages associated with the change of fuel. The predominant PAHs in TSPM include high molecular weight (HMW) congeners BghiP, DbA, IP, and BaP. The sum of 16 priority PAHs had a mean value of 72.7 ± 4.7 ng m^−3^. Potential sources of PAHs in aerosols were identified using diagnostic ratios and principal component analysis. The results reflect a blend of emissions from diesel and natural gas as the major sources of PAH in the city along with contribution from emission of coal, coke, and gasoline.

## 1. Introduction

Polycyclic aromatic hydrocarbons (PAHs) are comprised of carbon and hydrogen atoms in two or more aromatic rings [1]. They are a group of ubiquitous persistent organic pollutants possessing carcinogenic, mutagenic, and immuno-toxic properties [2]. They occur in the atmosphere due to emissions from gasoline- and diesel-powered vehicles and other sources such as coal, biomass, gas, and oil combustion [3–7]. The strainless arrangement of *sp^2^*-hybridized carbon atoms in six membered rings and, above all, the energy gain from the delocalized p-electrons, makes PAHs thermally very stable and therefore PAH are abundant molecules in the combustion zone. Therefore, they are always formed in combustion processes and are attributed to unburned, pyrolyzed, or partially oxidized fuel and lubricant oil that are transferred from the gas phase to the particulate phase by adsorption and condensation onto the existing particles or by nucleation of new particles when the exhaust cools [2].

PAHs occur in gaseous form and are adsorbed to particles in the atmosphere, depending on the volatility of the PAH species. Higher condensed molecules with four and more rings are particle-bound, whereas smaller PAHs mainly remain in the gas phase [8]. Once PAHs are released into the atmosphere, they are subjected to various atmospheric processes through which their dispersion, removal, transport and degradation can occur. Dispersion of atmospheric PAH is dependent not only on the magnitude of the emissions but also on the stability of PAH in the atmosphere [8]. PAH concentration is highly dependent upon the size of airborne particulate matter, with the greatest concentration being in the respirable size range [9].

PAHs have attracted much attention in the studies on air pollution recently because some of them are highly carcinogenic or mutagenic. In particular, benzo(a)pyrene has been identified as a highly carcinogenic [10]. The occurrence of PAH in urban air has caused particular concern because of the continuous nature of the exposure and the size of the population at risk. Humans can be affected by direct inhalation of polluted air, tobacco smoke, ingestion of contaminated and processed food and water, and through dermal contact.

PAHs have been less monitored in Asia, specifically in India, in comparison to its western counterpart. India is a developing nation which has experienced an increase in population and industrial expansion that has been accompanied by drastic increase in vehicular transportation. Information on the airborne concentration of toxic pollutants in developed countries is relatively abundant. However, for less developed nations as India, data are not routinely collected. In India, studies on measurements of PAHs in the ambient air of major cities are limited. Total PAH concentrations in ambient aerosols in Ahmedabad, Mumbai, Nagpur, Calcutta, and Kanpur show that PAH concentrations are 10–50 times higher than those reported internationally and range between 23 and 190 ng m^−3^ [11–15]. In an earlier study, it was reported that the annual average total PAH concentrations at four locations in New Delhi, which ranged from 150 to 1,800 ng m^−3^, were dominated by vehicular traffic [16]. Recently atmospheric concentrations of total PAHs were found to range from 1,049 to 1,344 ng m^−3^ at three sites in Delhi with higher concentrations in winter. Diesel- and gasoline-driven vehicles were identified as the principal sources of PAHs [17]. In addition, in the Indian urban environment, cooking fuel combustion is also a likely source of PAH. High concentrations of PAH have been measured in smoke from solid-fuel stoves burning wood, coal, and dried cattle manure [11], which along with kerosene stoves [18] are used as the primary cooking device by urban slum residents.

Agra, located in the state of Uttar Pradesh of northern India, is known for Taj Mahal, one of the wonders of the world. In addition to Taj Mahal, there are three more world heritage sites, namely, Agra Fort, Fatehpur Sikri, and the bird sanctuary at Bharatpur National Park. In addition, the Akbar's tomb at Sikandra in Agra (in proximity to Agra) and the Imtad-ud-Daulahs tomb in Agra are proposed as world heritage sites. The majority of industries in Agra comprise of foundries and several diesel generator manufacturing units. In foundries, the principal source of emission is cupola. The volume of gas exhausted and its concentration depends on the cupola, operations, melting rates, characteristic of charging material, and the coke [19]. Gases escape while drawing the hot metal and during casting. In the pit type of cupola, emissions are fugitive type. Besides a number of petha (a sweet) industries are operating in the city, which mainly use coal as fuel. There are also several sweet shops, pottery units that use coal, cow dung, and wood [19]. The vehicle fleet in Agra comprises of trucks, buses, delivery vans, taxis, private cars, and autorickshaws that are mainly diesel driven. The number of registered on-road diesel vehicles in Agra till January 2008 was approximately 51,938 [20]. Diesel-driven generators are almost exclusively used in the industrial units as well as in the households as a stand by to meet power requirements during periods of load shedding. The diesel quality used earlier was poor containing a high S content, to the extent of 0.25%, and a large amount of aromatics [19]. Moreover, a large population of the slum residents of the area use coal, coke, wood, kerosene, and cow dung cakes as the domestic fuel, which is burnt in small home made cook stoves.

The area in the immediate vicinity of the Taj Mahal in the form of a trapezoid is known as the Taj Trapezium Zone. It extends between 27°30′ N and 77°30′ E to 27°45′ N and 77°15′ E and 26°45′ N and 77°15′ E to 27°00′ N and 78°30′ E. In 1999, the Ministry of Environment and Forest, Government of India, has notified the Taj Trapezium Zone (Pollution Prevention and Control) Authority for protection and improvement of the environment in the Trapezium. In this connection several actions have been taken to control industrial and vehicular pollution. Industries have been encouraged to use natural gas instead of coal and there is strict vigilance on these industries not to use coal or coke; the brick kilns that were previously operational around the city have been closed. Likewise stringent actions have also been taken to control vehicular pollution. These include phasing out of grossly polluting vehicles plying within the city area and age limits for different categories of vehicles were fixed by Road Transport Authority (RTA). Diesel-driven autos fitted with particle traps are only given license and registration and to control emissions low sulfur (0.05%) diesel is made available. More and more CNG/LPG (compressed natural gas/liquefied natural gas) and battery-operated vehicles are being promoted. Similarly gas-driven electricity generator sets have been developed and their use has been encouraged. Simultaneously for the in-use vehicles strict checking of vehicular emissions is performed [19].

In the recent past we have conducted PAH measurement in total suspended particulate matter at Agra for short durations at different locations [7, 21]. These studies also revealed higher concentrations of PAH in TSPM that were mainly attributed to originate from vehicular traffic and combustion of coal and biomass comprising wood, crop residues, and agricultural wastes burnt in the domestic and industrial sector. In the present study results of PAH measurements carried out from May 2006 to December 2008 at a location close to the industrial area as well as the major highway (NH-2) crossing in the city are presented. An attempt has also been made to see the influence of the use of cleaner fuels through determination of sources of PAH by principal component analysis.

## 2. Materials and Methods

### 2.1. Description of Sampling Site

Agra is situated in the extreme southwest corner of Uttar Pradesh and stretches across 26°44′ N to 27°25′ N and 77°26′ E to 78°32′ E. Its borders touch Rajasthan to its west and south, the district of Firozabad to its East, and the districts of Mathura and Etah to its North. It is situated on the banks of river Yamuna, and it has a limited forest area supporting mainly deciduous trees. According to census 2001, the area of Agra district is 4027 km^2^ with a total population of about 1,316,177 and density about 21,148 of km^2^ with 386,635 vehicles registered and 32,030 generator sets. In Agra, 60% of NOx pollution is due to vehicles [20]. Three national highways (NH-2, NH-11, and NH-3) pass through the city. Like most cities of north India, the weather and climate of Agra is extreme and tropical. Agra suffers from extremities of climate with scorching hot summers and chilly winters. It is about 169 m above the mean sea level (msl) and has been reported [19] as having semiarid climate with atmospheric temperature in the range of 11–48°C (max) 0.7–30°C (min), relative humidity 25–95%, light intensity 0.7–5.6 oktas (cloudiness), and rainfall 650 mm per year. The climate of Agra has been broadly divided into three seasons: winter (October to February), summer (March to June), and monsoon (July to September). During monsoon period the temperature ranges between 26 and 39°C and relative humidity varies between 70 and 100%, respectively [19]. The winter months are cool and temperature ranges from 2 to 15°C while relative humidity is 60 to 90%. Summers are characterized by high temperature ranging from 23 to 45°C and low relative humidity 25 to 40%.

 Sampling was conducted on the roof of a single storeyed building situated in proximity to the industrial area as well as about 1.5 km from a busy roadway intersection situated on the National Highway Number2 (NH-2). NH-2 connects Delhi to Kolkata via Agra and is one of the busiest highways. On an average six to seven thousand vehicles ply on this highway and comprise about 3500 light motor vehicles (LMV), 500 light commercial vehicles (LCVs), and 2500 heavy commercial vehicles (HCVs) [22]. In this study total suspended particles (TSPs) were collected during May 2006 to December 2009 using a high-volume sampler. The samples were collected on predesiccated and preweighed glass fibre filters (Whatman, EPM 2000). Air was drawn at a flow rate of 1.1 m^3^ min^−1^ for 24 hr on an average. The accurate flow rate of the sampling device was determined by averaging the flow rates measured at the beginning and the end of the sampling period. Before bringing the samples to the laboratory, the filters were packed in aluminium foils to protect them from dust and were stored at low temperature to prevent the volatilization of low molecular weight PAHs.

The particulate matter contained in the filter was extracted thrice by using ultrasonic agitation with 200 mL dichloromethane for 1 hr after intervals of 10 min after every 15 min to ensure maximum recovery. All the three extracts were mixed together to make a composite sample. The extract was purified by means of cleanup in silica gel column to remove elements interfering in the analysis. The extract was transferred to the top of a glass column (10 cm × 1.0 cm i.d.) slurry packed with 2 g of silica gel (Fluka, 230 mesh). The column was eluted with dichloromethane to give fraction enriched with PAH. The PAH containing fraction was concentrated to 1.5 mL by a rotary evaporator and stored in Teflon vials at low temperature till analysis.

PAHs were analyzed in the splitless mode using a temperature gradient program [23] by gas chromatograph, (Shimadzu 17AATF, version 3.0) equipped with a FID detector and capillary column (25 m length, 0.3 mm internal diameter: BP) with dimethyl polysiloxane as stationary phase. Nitrogen was the carrier gas at a flow rate of 12.7 mL min^−1^. The oven temperature was held at 40°C for 5 minutes and programmed to rise to 179°C at 10°C min^−1^, held for 2 minutes, and then elevated to 300°C at 9°C min^−1^. The temperature of injector and detector was maintained at 210°C and 310°C, respectively. The GC was calibrated with a standard solution of 16 PAH compounds (Supelco EPA 610 PAH mixture). The procured PAH mixture contained the following 16 EPA priority PAHs in mixed solvent (methanol: dichloromethane; vol/vol, 1 : 1); Naphthalene (Nap), Acenaphthylene (Acy), Acenaphthene (Ace), Fluorene (Flu), Phenanthrene (Phen), Anthracene (Anth), Fluoranthene (Fla), Pyrene (Pyr), Benzo(a)anthracene (BaA), Chrysene (Chr), Benzo(b)fluroanthene (BbF), Benzo(k)fluoranthene (BkF), Benzo(a) pyrene (BaP), Dibenzo(a,h)anthracene (DbA), Benzo(ghi)perylene (BghiP), and Indeno (1,2,3-cd)pyrene (IP) in the range of 100 to 2000 *μ*g mL^−1^. Five point calibration curves for all the target analytes ranging were obtained by analysis of serial dilution of PAHs standard. Calibration curves were plotted by regression analysis. 1 *μ*L of the extracted sample was injected into GC and the program was set to run for 40 minutes. Individual PAH were identified by comparing their retention time with the standard chromatogram. Each PAH compound was quantified by plotting its peak area on the regression curve of standard. The limit of detection of the chromatographic method determined through serial dilution of the PAH standard varied between 0.007 and 0.16 ng for the different compounds. The limit of quantification (LOQ) defined as the limit of detection divided by the sampling volume [24] was in the range of 1.8 × 10^−7^ and 4.10 × 10^−5 ^ng m^−3^ [7]. The recovery efficiency of the method was evaluated by the analysis of filters spiked with known concentration of standard PAH compounds [7]. Most of the compounds provided high recoveries with mean values ranging between 70 and 80%. Field and laboratory blank were routinely analyzed for quality control. Blanks levels of individual analytes were normally very low and in most cases not detectable. Some PAH compounds like BaA and Chr, BbF and BkF, and DbA and IP coelute in the method followed in this study; hence these coeluting pairs have been reported as their sum [25].

## 3. Results and Discussion

### 3.1. PAH Concentrations and Seasonal Variations

Both TSP and PAH concentration presented a large variability with a skewed distribution. The geometric mean concentrations of TSP, individual PAH, and total PAH (i.e., sum of all determined PAH in each sample, TPAH) are listed in Table 1.

The mean concentration of individual PAH ranged from 0.9 to 20.5 ng m^−3^. PAH can be classified into low molecular weight compounds (Nap, Acy, Ace Flu, Phen, Anth, Fla and Pyr) and high molecular weight compounds (BaA, Chr, BbF, BkF, BaP, DbA, IP, and BghiP). The characteristic mass distribution pattern of PAH shows that the mass distribution in air was dominated by high molecular weight PAH with 5 to 6 rings such as BbF, BkF, DbA, IP, BaA, Chr, BaP and BghiP. PAHs are semivolatile organic compounds and occur in both gaseous and particulate phases in the atmosphere. The vapor-particle partition exhibits a strong dependence on molecular weight. High molecular weight compounds dominate in the particulate phase while low molecular weight PAHs reach higher concentrations in the vapor phase. The HMW PAHs are mainly derived from the vehicular emissions and are known to exist adsorbed on particulate matter owing to their low vapor pressure. Moreover, some of the higher molecular weight PAHs in the air are short lived; half life is about 2.4 h for BaP, 1–13 h for DbA and BaA, and 0.31–10 h for BghiPs, therefore, they are easily detected at sites where sampling is carried out close to emission sources [26]. Among the low molecular weight compounds Acy, Ace, and Anth are in higher concentrations than Pyr, Fla, Flu, and Phen. The sum of the concentration of nine major combustion PAHs (CPAH) (Fla, Pyr, BaA, Chr, *B*(*b* + *k*)*F*, BaP, IP, and BghiP) accounted for 77% of the total PAH mass. The ratio of CPAH/TPAH was 0.66 and is higher than the ratio reported for noncatalyst (0.41), catalyst (0.51) automobiles, and heavy duty diesel trucks (0.30) [27]. Noncatalyst vehicles emit 27 times more PAHs especially HMW PAHs than catalyst-equipped vehicles [27]. Less efficient emission control system in the vehicle fleet and more extensive combustion activities in the city may account for this high CPAH/TPAH ratio. Sum of the carcinogenic PAHs including BaA, BbF, BkF, BaP, DbA, and IP accounted for 64% of TPAH. IARC has classified total carcinogenic PAH as “*probably carcinogenic*” to humans (namely, BaA, BaP, and DbA) and as “*possibly carcinogenic*” to humans (namely, BbF, BkF, and IP) [28]. In the present study the “*probably carcinogenic*” compounds accounted for 38% while “*possibly carcinogenic*” compounds accounted for 26% of total PAH. The carcinogenic PAH have high molecular weights and are especially bound to suspended particles. BaP has been the most extensively measured PAH in urban areas around the world due to its high carcinogenic property. Average BaP concentration at the site was 12.3 ng m^−3^, that is, less than the permissible exposure limit (0.2 mg m^−3^) of PAH set by the Occupational Safety and Health Administration (OSHA) [29].

Atmospheric PAH levels can differ to an extent of 40% depending on the sampling system, extraction solvents, analytical techniques, and detectors [30]. PAHs determined in airborne particles are also influenced by regional climatic and source characteristics [31]. A comparison has been made with other Indian sites in Table 2.

PAH concentrations varied inversely with temperature (*r* = −0.65, *P* = 0.05), that is, higher concentrations in the cold months and lower ones in the warm months. The winter-to-summer ratio of individual compounds varied between 1.14 (DbA) and 2.65 (IP). The increase in particulate PAH concentration during the winter and the dependence of PAH concentration on atmospheric temperature have been reported in a number of studies [35–38]. Source emissions and meteorological conditions as well as gas particle partitioning may result in winter and summer difference of PAHs concentrations [39]. Reduced atmospheric dispersion resulting from lower mixing height as well as reduced atmospheric reaction can lead to higher pollutant concentrations in ambient air during winter. Low atmospheric temperature can affect the distribution of PAHs between the gas and particle phases and result in a relatively larger portion of PAH partitioning to the particle phase in winter. In contrast during the summer a higher ambient temperature could change the distribution of PAHs between the gaseous and particulate phases by increasing the vapor pressure of pollutants that adhered to atmospheric aerosols and favoring the volatilization of PAHs from the particulate to gaseous phase. It is also well known that PAHs in the atmosphere are subjected to photochemical or thermal reactions with ozone, nitric oxides, and hydroxyl radicals that lead to PAHs degradation, especially in the warmer seasons [40]. High solar radiation could enhance the reaction of volatile organic compounds in forming ozone active hydroxyl radicals that in turn react with PAHs and reduce their concentrations [41]. In addition to temperature effects on the physicochemical property of atmospheric PAHs, anthropogenic factors can also lead to seasonal variation of particulate PAHs. During the cold season, PAH emissions from automobile exhaust are higher because of low ambient temperature and increased cold start impacts [42]. Another reason for the higher concentrations in winter could be due to an increase in the emissions owing to the fossil fuel usage for space heating purposes. Sharma et al. [17] have also found winter/summer ratios of total PAH between 2.41 and 3.69 at three sites in Delhi. However PAHs in winter have been found higher by a factor of 1.5–10 than that in summer in studies carried out in Europe and the USA [8, 43–45]. Similarly, Guo et al. [46] found high winter/summer ratios of 8.6 and 7.5 at two sites in the city of Hong Kong.

The contribution of the combustion-derived PAH (CPAH) to total PAH also varied between the two seasons. Their contributions were 64 and 72%, respectively, in summer and winter. The lower contribution is summer meant that vehicular emissions may be a contribution in summer while in the winter season, emission sources were more complex.

### 3.2. Diagnostic Analysis and Principal Component Analysis

Studies indicate that particulate organic samples collected in tunnels are enriched in benzo(ghi)perylene and coronene, which are characteristic of gasoline engines [47]. Diesel exhaust is found to be enriched in Fla, Chr, and Pyr [48] while Anth, Phen, Fla, and Pyr have been identified as source fingerprints of wood combustion and BaA and Chr as markers of coal combustion [49]. The concentrations of these marker compounds and their ratios can give some indication about the impact of different sources of airborne compounds and can be used in distinguishing emissions [49, 50]. In this study, the following PAH concentration diagnostic ratios characteristic of the anthropogenic emissions were calculated: IP (IP + BghiP), BaA/(BaA + Chr), BaP/(BaP + Chr), BghiP/IP, Phen/(Phen + Anth), BaA/BaP, and BbF/BkF. A comparison between the various diagnostic ratios obtained in this study with the standard values reported in the literature is shown in Table 3. Analysis of the ratios could be associated to various sources. The IP/(IP + BghiP) ratio is found to be 0.72, which is similar to that reported for diesel and gasoline emissions [27, 44, 46]. Similarily the value of BaA/(BaA + Chr) ratio has been reported to vary between 0.38 and 0.64 for diesel emissions and between 0.22 and 0.5 for gasoline emissions [51, 52]. For emissions from coal burning this ratio has been observed to be 0.50 [53]. In the present study this ratio is 0.44 which can be attributed to vehicular (diesel and gasoline) emissions. The vehicular influence can further be assessed from Phen/(Phen + Anth) ratio whose value of 0.68 is comparable to values reported for diesel, coal, and crude oil burning and gasoline emissions [49, 51]. The ratio BaP/(BaP + Chr) with its value 0.61 again indicates towards contributions from diesel emissions. The BaA/BaP and BghiP/IP ratios of 0.50 at this site may be inferred to the contribution from gasoline emissions [50]. Further BbF/BkF ratio with a value >0.5 may be attributed to diesel emissions [10]. These results suggest the influence of multiple sources like the burning of fuels, such as diesel oil, gasoline, and coal. Thus diagnosis of ratios contributes quantitatively to the identification of the main emission sources in the studied area. It is to be noted that the diagnostic ratios method should be used with caution because it is often difficult to discriminate between some sources [4]. For example, a ratio of 0.62 for IP/IP + BghiP has been suggested for wood burning [54] whereas a value between 0.35 and 0.70 indicates diesel emissions [55]. Hence, it would be difficult to differentiate diesel emission from biomass emission based only on one proposed diagnostic ratio. Recently Galarneau [56] has reviewed the caveats associated with the use of diagnostic ratios in source apportionment of PAHs in ambient air. The validity of this approach rests on the assumption that each source or source type is associated with relative proportions of the compounds that are unique and secondly the relative proportions of the compounds are assumed to be conserved between the emission source and the receptor or the points of measurement. These assumptions hold for PAH under limited set of conditions [56]. These ratios can also be altered due to the reactivity of some PAH species with other atmospheric species like ozone and/or oxides of nitrogen or by the volatility and solubility of some PAH [4, 57]. To minimize this bias, the diagnostic ratio of PAH with similar physicochemical properties should be used. Degradation processes during the sampling process may also modify PAH concentrations and thus the ratios between PAHs.

In addition to the diagnostic ratios between PAH, Principal Component Analysis (PCA) was used as an exploratory tool as it enhances the accuracy of emission source identification by selecting statistically independent source tracers [46]. This method segregates measured ambient concentrations according to groups of covarying compounds and comparing these groups to suspected source profiles. Thus using PCA, it is possible to simplify the interpretation of complex systems and to reduce the set of variables to few new ones, called factors. Each of these factors can be identified as either an emission source or a chemical interaction by the most representative compounds. Some PAH compounds and their combinations are frequently used as source markers such as Flu, Chr, and Pyr for diesel exhaust [46, 58], Flu, Pyr, BbF, and BkF for heavy duty diesel vehicles [4, 59], BghiP for gasoline vehicles [4, 58, 60], Flu and BaP [31] or Ant, Phe, Flu, and Pyr [4, 5, 31, 49] or NaP and Ant [49] for wood combustion, Ant, Phe, Flu, Pyr, BaA, and Chr for coal combustion [4, 5, 49, 60], Phe and Flu [4, 49] for coke production, Flu and Pyr [4, 61] for oil burning, and Ant, Phe, BaP, BghiP, and Chr [4] for steel industry emissions.

Before subjecting the data to PCA, a pretreatment involving an autoscaling of the data was performed. In this autoscaling procedure all variables were mean centered and scaled to unit variance, by subtracting the mean *x*_*j*_ of each variable *j* from the result *x*_*ij*_ of each sample *i* for that variable and dividing the result by the standard deviation *s*_*j*_ of that variable. The resulting autoscaled *z*_*ij*_ were used instead of the original *x*_*ij*_:

(1) $$z_{ij}\;=\frac{\left(x_{ij}-x_{j}\right)}{s_{j}}.$$

**Figure 1:**
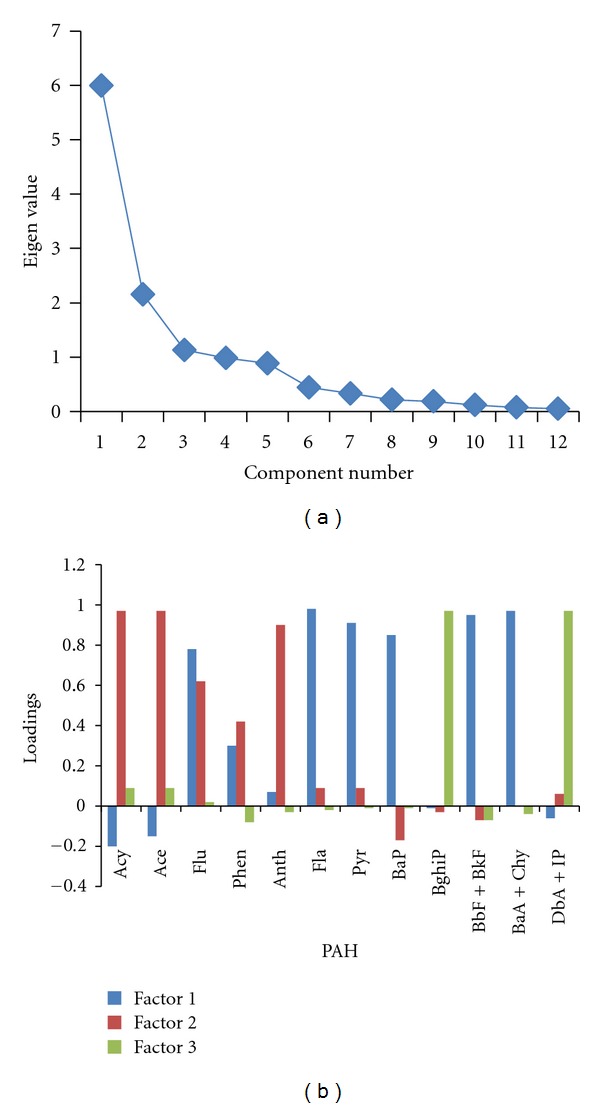
(a) Scree plot. (b) Factor profile.

As a result of this autoscaling, each variable has about the same range and it is avoided that some variables would be more important than others because of scale effects. In the present study, PCA was attempted by SPSS (version 10.0) by adopting Varimax procedure for rotation of factor matrix into one that was easier to interpret. Deciding the number of factors to retain for each data set is a critical issue. Retaining too few factors results in combining different sources, while retaining too many factors splits sources among factors in a physically unreasonable manner. The number of factors to be retained was decided on the basis of the breaks in the slopes of scree plots that are plots between eigen values and PCs as shown in Figure 1(a). PCs with eigen values greater than 1were retained as they provided a reasonable physical interpretation of sources. The PCs were interpreted on the basis of their loadings. Factor loadings greater than 0.5 were considered statistically significant. The factor profiles were constructed on the basis of factor loadings. These are shown in Figure 1(b). Three factors were obtained. They explained approximately 80% of the total variance of data. Factor 1 was associated with BaA + Chr, Fla, BbF + BkF, BaP, Flu, and Pyr and accounted for 44% of the total variance.

This factor was attributed to industrial emissions (stationary source). BbF and BkF are indicators of diesel exhaust emissions mainly emitted from diesel generators that are frequently operated during power cuts in the various industrial units located in this area and partly also contributed by heavy duty diesel vehicles plying around the area. Fla, Pyr, BaP, and BaA + Chr in this factor can be associated to emissions from natural gas combustion. BaA, Chr, and BaP are associated with combustion of natural gas [16, 27, 52] and BaA has been considered as a tracer for this source [52]. The presence of Chr with BaA, Fla, and Pyr in natural gas combustion has also been reported by Daisey et al. [62]. Factor 2 explains 22% of the total variance and includes Acy, Ace, Flu, Phen, and Anth. This factor can be attributed to coal and wood combustion. Khalili et al. [49] identified that Ant, Phe, Fla, and Pyr were source fingerprints of wood combustion, while the third factor explaining 14% of the variance can be associated to emissions from gasoline vehicles showing higher loadings on BghiP and DbA + IP.

### 3.3. Health Risk Assessment

An essential prerequisite for determining the human health risk associated with PAH in the environment is accurately characterizing the toxicities of individual PAH. As the toxicities of individual PAH differ considerably, toxicity assessments of PAH are complex [63]. One approach commonly referred as the Toxic equivalence Factor (TEF) calculates the inhalation risk for excess lung cancer over the risk posed by BaP for each of its copollutant carcinogenic PAH in the polluted ambient air [64]. TEFs are toxicity potency factors that are used by scientists and regulators globally as a consistent method to evaluate the toxicities of highly variable mixtures of PAH compounds. TEF was initially developed for estimation of the risk caused by complex mixtures, such as dioxins, and could help to characterize more precisely the carcinogenic properties of complex mixtures. The method relates individual compound toxic potency to a compound, often the most toxic compound in the mixture [65]. A few proposals for TEFs are available [66–70]; among these the list of TEFs completed by Nisbet and LaGoy [69] reflects well the actual knowledge of the toxic potency of each individual PAH compound [70]. In the PAH family, BaP the most toxic member, is assigned a TEF of one. According to this approach the health risk assessment of PAHs can be assessed based on its BaP equivalent concentration (BaPeq). BaPeq concentration for each PAH is calculated by multiplying its concentration with the corresponding TEF. The carcinogenic potency of total PAHs can be assessed by the sum of the BaPeq concentration of each PAHs. The TEFs-adjusted concentrations of each PAH along with their proposed TEFs are presented in Table 4. The total BaPeq concentration was found to be 27.6 ng m^−3^. The LMW compounds show only a negligible contribution towards carcinogenicity. The major contributor is BaP followed by BghiP. This underlines and confirms the importance of BaP and BghiP as a surrogate compound for PAH mixture in ambient air. The carcinogenic PAHs (BbF, BkF, BaP, BghiP, and IP) accounted for 97% of the total BaPeq concentrations which is similar to 97% and 93% in Ho Chi Minh city, Vietnam, and Osaka, Japan, respectively [58]. These results imply that particle bound PAHs with high molecular weight play an important role on total BaP eq concentration from health risk point of view. Other compounds like Acy, Ace, Anth, Pyr, BaA, and Chr which also dominate in the PAH mixture obviously play a minor role in the carcinogenicity of the PAH mixture in ambient air.

## 4. Conclusion

In this study, TSP samples were collected and quantified for 16 PAH compounds. Mean total PAH concentration was found to be 72.7 ± 4.7 ng m^−3^ that is lower than the permissible exposure limit (0.2 mg m^−3^) of PAH set by the Occupational Safety and Health Administration (OSHA). The high molecular weight PAHs, namely, BghiP, DbA, IP, and BaP were the most abundant. Average BaP concentration was 12.3 ng m^−3^. Concentrations of Total PAHs as well as the individual compounds were lower in summer than in winter. Diagnostic ratios and principal component analysis indicated that the influences of vehicular sources and stationary sources involving both diesel and natural gas combustion were the principal sources while emissions from coal-, coke-, and gasoline-driven vehicles had a minor contribution.

## Figures and Tables

**Table 1:** Mean and standard deviations for all PAHs (ng m^−3^).

	Average	Standard deviation (ng m^−3^)
Naphthalene (NaP)	0.9	1.7
Acenaphthylene (Acy)	1.7	4.7
Acenaphthene (Ace)	1.8	1.2
Fluorene (Flu)	1.0	0.7
Phenanthrene (Phen)	0.9	0.2
Anthracene (Anth)	2.2	1.3
Fluoranthene (Fla)	0.9	1.3
Pyrene (Pyr)	3.1	2.3
Benzo(a)anthracene + Chrysene (BaA + Chr)	14.1	4.7
Benzo(b)fluoranthene + Benzo(k)fluoranthene (BbF + BkF)	20.5	6.8
Benzo(a)pyrene (BaP)	12.3	6.0
Dibenz(ah)anthracene + Indeno (123-cd) pyrene (DbA + IP)	17.3	3.1
B(ghi)perylene (BghiP)	11.1	6.2
TPAH	72.7	4.7
TSPM*	348	31.5
CPAH	47.6	—
CPAH/TPAH	0.66	—
Total Carcinogenic PAH	64%	—

*μg m^−3^.

**Table 2:** Comparison of total PAHs (ng m^−3^) with various cities of India.

Total PAHs	Range	City	Substrate	Extraction	Analysis	Reference
*∑*11 PAHs (PM_2.5_)	326–791 (Mean at 4 Sites)	Chennai	PTFE membrane Filters	DCM : Methanol (60 : 40); Ultrasonication	HPLC (Fluorescence Detector)	[32]
*∑*9 PAHs (PM_2.5_)	202.6–333.7 (Mean at 4 Sites)	Tiruchirappalli	PTFE membrane Filters	DCM : Methanol (60 : 40); Ultrasonication	HPLC (Fluorescence Detector)	[32]
*∑*11 PAHs (PM_10_)	8–97.9 (Mean at 4 Sites)	Agra	Glass fibre filter	Methylene Chloride; Soxhlet Extraction	GC-MS	[33]
*∑*12 PAHs (TSP)	1049–1344 (Mean at 3 Sites)	Delhi	Glass fibre filter	Toluene; Ultrasonication	GC-FID	[17]
*∑*12 PAHs (TSP)	672 (Mean)	Delhi	Glass fibre filter	Toluene; Ultrasonication	GC-FID	[34]
*∑*16 PAHs (TSP)	72.7 (Mean)	Agra	Glass fibre filter	DCM Ultrasonication	GC-FID	Present Study

**Table 3:** Diagnostic ratios.

	*Present study*	Diesel	Gasoline	Wood	Coal	Other sources	References
IP/(IP + BghiP)	*0.72*	0.37		0.62	0.56		[54]
		0.35–0.7	0.18				[53, 54]
			0.21–0.22				[27]
					0.33		[49]
							[53]

BaA/(BaA + Chy)	*0.44*	0.38–0.64					[52]
			0.22–0.55				[52]
					0.50		[53]

BaP/(BaP + Chy)	*0.61*	>0.73	0.49				[46, 55]

Phen/(Phen + Anth)	*0.68*	0.65	0.5		0.76	>0.70 (Crude oil)	[51, 55]

BaA/BaP	*0.50*	0.90–1.70	0.50–0.70	1.0–1.5			[50]

BbF/BkF	*1.08*	>0.5					[52]

**Table 4:** Proposed Toxic Equivalency Factors (TEFs) for individual PAHs and calculated TEF-adjusted concentrations of measured samples.

PAH	TEF (data from [69])	TEF-adjusted concentrations in ng m^−3^ (Present Study)
Naphthalene (NaP)	0.001	0.0009
Acenaphthylene (Acy)	0.001	0.0017
Acenaphthene (Ace)	0.001	0.0018
Fluorene (Flu)	0.001	0.0010
Phenanthrene (Phen)	0.001	0.0009
Anthracene (Anth)	0.01	0.0223
Fluoranthene (Fla)	0.001	0.0002
Pyrene (Pyr)	0.001	0.0031
Benzo(a)anthracene (BaA)	0.1	0.616
Chrysene (Chr)	0.01	0.079
Benzo(b)fluoranthene Benzo(k)fluoranthene (BbF + BkF)	0.1	1.064
Benzo(k)fluoranthene (BkF)	0.1	0.984
Benzo(a)pyrene (BaP)	1	12.27
Indeno(123-cd)pyrene (IP)	0.1	1.479
Dibenz(ah)anthracene (DbA)	1	0.023
B(ghi)perylene (BghiP)	0.01	11.13
